# Some Newspaper Surgery

**Published:** 1900-12

**Authors:** 


### SOME NEWSPAPER SURGERY.

DR. W. W. KEEN, of Philadelphia, has been having a hard time with certain newspapers in his city, which persisted in publishing reports of his clinic without his authority and in a most glaring inaccurate and ludicrous manner. Not only this, but the tone of the articles was distinctively offensive. Dr. Keen wrote the newspaper requesting that nothing be published concerning him or his clinic. The reply to this was the publication of another article as offensive as the first and more ludicrous if such a thing were possible. Dr. Keen then appealed to the proprietor of the newspaper and secured the relief he asked for. That is a case of honesty of purpose for which Dr. Keen will be heartily commended by every surgeon of standing in the country. Quite different was the case in Buffalo within the past two weeks when an article was published in a morning newspaper describing an operation under the newest fad in anesthesia —cocainisation of the spinal cord. This article has all the ear marks of having been either written or dictated by a medical man, and an idea of the general literary excellence and scientific accuracy may be had by reading this paragraph:

> The patient was first placed in a sitting position on the operating table. His body was inclined forward at an angle of about 45 degrees. Then the operating surgeon inserted between the articulation of the fourth and fifth vertebrae a hypodermic point 2½ inches long. The point was charged with fifteen minimums of cocaine.
>
> In the method adopted, the bare nerve or cord is reached by the use of a long hypodermic point. Then the dose of cocaine is directed immediately into the cord. The circulation in and about the cord is very sluggish and the opportunity for the absorption of the cocaine into the general circulation is very small, as there is less danger in using a large dose in the medullary canal than there would be in fusing a small dose into the general circulation.

This bears out what the JOURNAL has often said before, that a newspaper which wishes to print medical news should have an educated physician on its staff. Until newspapers adopt this plan they will continue to make odd spectacles of themselves.

